# Social isolation, physical inactivity and inadequate diet among European middle-aged and older adults

**DOI:** 10.1186/s12889-021-10956-w

**Published:** 2021-05-15

**Authors:** Alice Delerue Matos, Fátima Barbosa, Cláudia Cunha, Gina Voss, Filipa Correia

**Affiliations:** 1 Department of Sociology, Institute of Social Sciences, University of Minho, Braga, Portugal; 2 Communication and Society Research Centre, Institute of Social Sciences, University of Minho, Braga, Portugal

**Keywords:** Social isolation, Physical inactivity, Inadequate diet, Middle-aged and older adults, Europe

## Abstract

**Background:**

Social isolation is a growing public health concern for older adults, as it has been associated with poor health and premature mortality. On the other hand, physical inactivity and an inadequate diet are important health risk behaviours associated with physical and mental health problems. Considering that there is no research examining the possible relationship between social isolation and the above mentioned health risk behaviours of European middle-aged and older adults, this cross-sectional study aims to contribute to filling this gap.

**Methods:**

We used data from the SHARE project (Survey of Health, Ageing and Retirement in Europe), wave 6 (2015), release 7.0.0 (*N* = 67,173 individuals from 17 European countries plus Israel). Statistical tests for a two-group comparison were carried out to assess the differences between highly socially isolated individuals and low/intermediate socially isolated ones. Logistic regressions by country were performed to examine whether social isolation is associated with physical inactivity and an inadequate diet in the population aged 50 + .

**Results:**

Our results point out that, for the majority of the countries analysed, highly socially isolated individuals are more likely than low/intermediate isolated ones to be physically inactive and to consume less fruit or vegetables on a daily basis. In 9 European countries (Austria, Germany, Sweden, Denmark, Greece, Belgium, Poland, Luxembourg and Estonia) highly socially isolated individuals are more likely to be physically inactive. On the other hand, in 14 European countries (Austria, Germany, Sweden, Italy, France, Denmark, Greece, Switzerland, Belgium, Czech Republic, Luxembourg, Slovenia, Estonia and Croatia), high social isolation increases the likelihood of having an inadequate diet.

**Conclusion:**

Highly socially isolated European middle-aged and older adults are more prone to be physically inactive and to have an inadequate diet in terms of daily consumption of fruit and vegetables. The reduced social integration, social support and companionship of the highly socially isolated individuals may explain this association. Our results reinforce the importance of social and health policies targeting highly socially isolated European individuals aged 50 + **.**

## Background

Social isolation is a growing public health concern [1, 2] and a major health problem for older adults living in the community [3]. By social isolation, we mean the lack of social contacts, as stated by Holt-Lunstad et al. [2]. More specifically, social isolation is characterized by few relationships with other people, as well as little involvement in social organizations [4, 5]. Previous studies have shown that social isolation is linked with health risks that are especially harmful for older adults [6], and associated with more chronic conditions, functional impairment, worse mental and cognitive health, as well as an increase in premature and all-cause mortality [2, 7–12]. According to Blazer [13], social isolation is a strong source of stress that contributes to several diseases that might lead to morbidity and mortality. Additionally, social isolation is part of a cascade of complex psychosocial factors that interact to cause negative health outcomes in older adults [3].

A recent report shows that 18% of European adults (75 million people) are socially isolated, but their distribution by country is very uneven [5]. Moreover, the literature indicates that social isolation is more prevalent in older people [14]. Health problems [15, 16], retirement, the break with support networks and friends [17], economic constraints and the death of partners and friends [18] are associated with social isolation among older people.

Low levels of physical activity and the inadequate intake of fruit and vegetables are important risk factors for health [19]. Physical inactivity is responsible for 13.4 million disability-adjusted life-years worldwide [20] and is more common in older age groups [21, 22]. Some studies [22, 23] point out that there is considerable variation in the prevalence of the phenomenon between countries. According to Gomes et al. [22], the prevalence of physical inactivity in the European population aged 55+ ranges from 4.9% in Sweden to 29% in Portugal.

The literature shows that, among older European adults, a higher consumption of fruit and vegetables is correlated with improved overall health (physical health, mental health, physical functioning and cognitive health) and slower disablement processes [24]. On average, in the Organization for Economic Cooperation and Development (OECD) countries, 57.1% of adults consume fruit and 59.6% have vegetables in their diet on a daily basis [25], but there are significant differences between these countries regarding the daily intake of fruit and vegetables.

Inconsistencies regarding the relationship between age and the consumption of fruit or vegetables have been reported. While some studies indicate that older individuals consume less fruit and vegetables [26, 27], one study finds that individuals aged 65+ consume more fruit and vegetables per day compared to younger ones [28].

Some studies highlight the association between social isolation, on the one hand, and lower physical activity and sedentarism [4, 14, 29, 30], and a higher risk of malnutrition and dietary inadequacy, such as low consumption of fruit and vegetables [1, 14, 29, 31, 32], on the other.

In England, studies [4, 29] reveal that socially isolated individuals aged 50 and plus have less physical activity and greater sedentary time. In the same line, Hämmig [14] found that, independently of their age, socially isolated people living in Switzerland have high risk of physical inactivity. Moreover, individuals living in Northern Manhattan community (United States of America), with markers of social isolation have lower levels of physical activity [30].

Regarding to higher risk of malnutrition and dietary inadequacy, studies developed in Switzerland, England, Czech Republic and Poland [1, 14, 29, 31, 32] show that socially isolated individuals have high risk of poor or inadequate diet, not consuming the desirable quantities of fruit and vegetables.

However, despite the relevance of the above-cited studies, it is unknown whether the relationship between social isolation, on the one hand, and the mentioned health risk behaviours, on the other, remains constant in a very heterogeneous Europe. Thus, this study aims to fill a gap in scientific knowledge by examining this relationship in a wide range of countries with very different levels of social isolation and prevalence of physical inactivity and inadequate diet.

## Methods

### Study population

This research uses data from the SHARE project (Survey of Health, Ageing and Retirement in Europe), wave 6 (2015), release 7.0.0 (10.6103/SHARE.w6.700) [33] (*N* = 67,173). SHARE is a European multidisciplinary and cross-national panel database of microdata on health, socioeconomic status and social and family networks [34].

The SHARE target population consists of everyone aged 50 years and over with their regular domicile in a SHARE country. SHARE uses representative samples of the 50+ population in each European country, plus Israel. Data is collected in face-to-face interviews using the computer-assisted personal interviewing (CAPI) method. Proxy interviews are allowed when respondents are unable to participate in the survey, such as health reasons. For further methodological details of the SHARE project, please see Börsch-Supan et al. [34]. In wave 6, 17 European countries (Austria, Belgium, Switzerland, Germany, Denmark, Spain, France, Greece, Italy, Sweden, Czech Republic, Poland, Estonia, Portugal, Slovenia, Luxembourg and Croatia) and Israel participated in the SHARE project.

Our study includes middle-aged and older adults from the above countries, in a total of 67,173 individuals aged 50+. The age criteria adopted in this study derives from the fact that we want to capture three distinct periods of the life course (pre-retirement, post-retirement and oldest age) [35] that might influence physical activity and dietary behaviours.

Within our sample, 44% individuals are men and 56% are women.

### Ethics

The SHARE study is guided by international research ethics principles, such as the Respect Code of Practice for Socio-Economic Research and the ‘Declaration of Helsinki’ [36]. SHARE wave 6 was reviewed and approved by the Ethics Council of the Max Planck Society.

### Measures

Physical inactivity and the non-consumption of fruit or vegetables every day are our dependent variables. Approaches to measure physical inactivity vary across studies. In this research, physical inactivity is defined as never or almost never engaging in moderate (i.e., activities requiring a low or moderate level of energy such as gardening, cleaning the car, or walking) or vigorous physical activity (i.e., sport, heavy housework, or a job that requires physical labour). SHARE respondents were asked how often, in their daily life, they engaged in vigorous activity (i.e., sport, heavy housework, or a job that requires physical labour) or moderate activity (i.e., activities requiring a low or moderate level of energy such as gardening, cleaning the car, or walking), with four response options: 1. more than once a week; 2. once a week; 3. one to three times a month; 4. hardly ever, or never. Based on the definition of physical inactivity by Gomes et al. [22], individuals who answered “one to three times a month” and “hardly ever, or never” to both questions were considered physically inactive.

The variable non-consumption of fruit or vegetables every day was built using the question “How often per week do you consume a serving of fruits or vegetables?”, with the following response answers: 1. Every day; 2. 3–6 times a week; 3. Twice a week; 4. Once a week; 5. Less than once a week. Individuals who do not consume a serving of fruit or vegetables every day are those who answered that they only consume it 3–6 times a week, twice a week, once a week and less than once a week.

Social isolation is the independent variable of interest on our design model. We used the construct of social isolation proposed by Shankar et al. [37] that is based on five conditions: not living with a partner (scored as 1), not belonging to any organisations, clubs or religious groups (scored as 1) and having less than monthly contact with friends, family or children (scored as 1 each). The score ranged from 0 to 5, with higher scores meaning higher levels of social isolation. According to Shankar et al. procedures [37], individuals with a score of zero are classified as having a low level of social isolation, individuals with a score of one as having an intermediate level of social isolation and individuals with a score of two or more as having high levels of social isolation. As has been done in other recent researches [29, 38, 39], in this study we grouped individuals with scores of 0 and 1 into a first category and individuals with scores of 2 or higher into a second category. Based on the literature review, the current research includes several sociodemographic, economic and health co-variables. Sociodemographic variables are: age at the time of interview; sex; educational level classified according to the International Standard Classification of Education (ISCED-97). ISCED-97 is divided into three categories: primary schooling or less, secondary education, and post-secondary education [40].

The economic situation of the respondent is analysed through the total of household net income that is adjusted for purchasing power parity and household size square root and divided into tertiles (lowest, middle and highest).

**Fig. 1:**
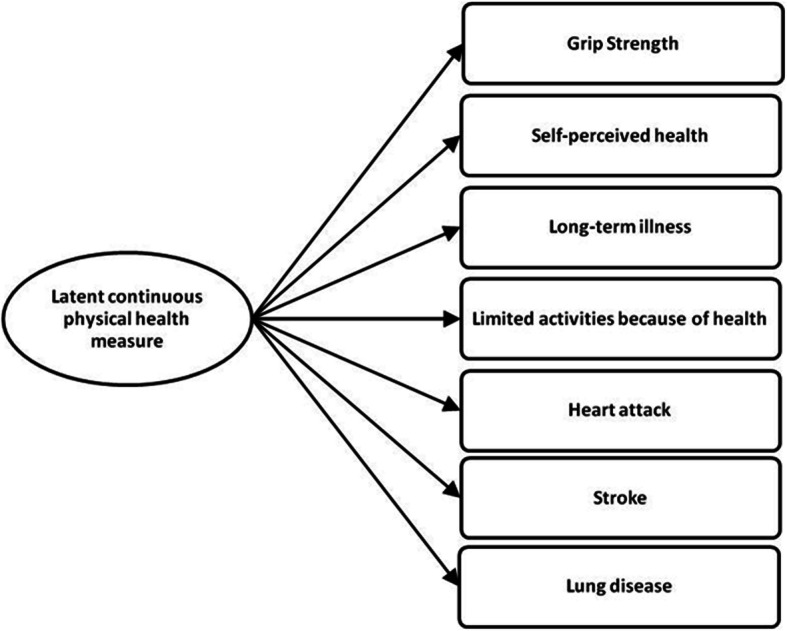
Latent continuous physical health measure. Source: Authors’ construction, based on Ploubidis and Grundy [41] and Di Gessa et al. [42] procedures

Our model controls for several health, and health risk behaviour variables: physical and mental health measures, alcohol and tobacco consumption, and doctor’s appointments. Based on Ploubidis and Grundy [41] and Di Gessa et al. [42] procedures, we used a Confirmatory Factor Analysis (CFA) to created a latent continuous physical health measure that was introduced in the model as co-variable. In SHARE, this measure combines an objective health indicator (maximum grip strength, using one or both hands) and six subjective ones (Fig. 1). The first subjective variable used is self-perceived health using a 5-point ordinal scale (poor, fair, good, very good or excellent). The second subjective variable of the physical health measure is the presence of long-term illness. The third subjective measure focuses on limitations in carrying out activities because of health problems, coded as being severely limited, limited, but not severely and not limited. The other subjective variables of the physical health measure are: having had a heart attack, having had a stroke, and having had chronic lung disease.

According to Ploubidis and Grundy [41], physical health measures are less subject to measurement error and have greater repeatability and reliability compared to individual health indicators used separately. The F-scores ranges from − 1.91 to 1.42, with higher scores representing better health. To build this variable we used MPLUS, version 7, WLSMV estimator (Muthén & Muthén, 1998–2012) and our model revealed a good model fit: Root Mean Square Error of Approximation (RMSEA) is 0.032; Comparative Fit Index (CFI) is 0.984 and Tucker-Lewis Index (TLI) is 0.976.

Mental health was assessed by the EURO-D 12-item scale [43] that takes into account 12 questions about feelings of depression, pessimism, wishing death, guilt, irritability, tearfulness, fatigue, sleeping troubles, loss of interest, loss of appetite, reduction in concentration, and loss of enjoyment over the last month. This scale ranges from 0 to 12. Clinically significant depression symptoms are defined according to Dewey and Prince [44] procedures: when the EURO-D score is greater than three, there are clinically significant depression symptoms.

Excessive alcohol consumption is measured by asking the question “During the last 7 days, how many units of alcoholic beverages did you have in total?”. The same categories as in Cerdá, Johnson-Lawrence and Galea [45] were used. The results took the respondents’ sex into account based on the criterion that excessive alcohol consumption for men means drinking more than 21 units of alcohol in the last 7 days, and more than 14 units for women.

Finally, our design model considers whether a respondent had ever smoked daily and the number of doctor’s appointments in the last 12 months.

### Analysis

This cross-sectional study was developed in several stages. Firstly, we performed a missing data analysis per country and we found missing values higher than 5% in some economic and health variables. Following Jakobsen et al. procedures [46], that refers that multiple imputation should be used with proportions of missing data higher than 5%, and since SHARE provides multiple imputations of the missing values, we used imputed economic and health variables in order to maximize the number of observations. After the inclusion of these imputed variables, missing data were residual (lower than 1%, per country).

Secondly, in order to assess the differences in characteristics of the study population, statistical tests for a two-group comparison (t-test (t) and chi-square tests (X^2^)) were carried out. The highly socially isolated individuals were compared with low/intermediate socially isolated ones. To complement these analyses, effect size measures (Cohen’s d/Phi) and Confidence Intervals (CI) for these effect size measures were calculated. Thirdly, the percentages of individuals aged 50+ who were physically inactive and did not consume fruit or vegetables every day were analysed according to social isolation, by country. Lastly, in order to analyse if social isolation is associated with physical inactivity and an inadequate diet in the population aged 50+, we performed logistic regressions, by country. The Model 1 (association between social isolation and physical inactivity) was adjusted for several confounders: age, sex, education, income, physical and mental health, excessive alcohol consumption, having ever smoked, number of doctor’s appointments in the last month and non-consumption of fruit or vegetables every day. The Model 2 (association between social isolation and inadequate diet) was adjusted for all previously mentioned variables except non-consumption of fruits and vegetables every day, plus physical inactivity.

Considering the potential presence of endogeneity of the social isolation variable in our logistic regressions, which might invalidate the analysis interpretations, we used the method Two Stage Residual Inclusion (2SRI) [47]. The instrumental variable *household size* was used. In order to test the weakness of the instrumental variable, a *weak instrument test* was performed by country and the *rule-of-thumb* measure was used [48]. As all our results were higher than 10, we assumed that our instrument is robust. To test the presence or absence of endogeneity, the *Hausman Test* was performed, by country [49]. Through the *Hausman Test* results, we were able to confirm the absence of endogeneity in our logistic regressions.

Statistical analyses were performed using IBM SPSS 25 [50] and software R 4.0.2 [51].

## Results

Table 1 displays the characteristics of the study population according to social isolation level. Firstly, individuals with low/intermediate levels of social isolation were compared with those with a high level of social isolation. Statistical tests for a two-group comparison showed that there are significant differences between the group of individuals with low/intermediate social isolation and the group with a high level of social isolation for all the analysed variables. Overall, the group of highly socially isolated individuals are older (70.1 years compared to 65.1 years), predominantly women (67.6% compared to 50.3%), less educated (54.3% primary or less education compared to 38.3%) and report lower income (39.4% compared to 31.9%) than the individuals with low/intermediate levels of social isolation. The group of highly socially isolated individuals showed lower levels of physical health (− 0.22 compared to 0.04), higher percentages of depression (43.1 compared to 26.7) and a higher number of visits to the doctor (7.7 compared to 6.7). The group of highly socially isolated individuals showed lower percentages of excessive alcohol consumption (3.2% in comparison with 4.2% of individuals with low/intermediate social isolation) and smoking (43.4% in comparison with 47% of individuals with low/intermediate social isolation). Finally, the group of highly socially isolated individuals was more physically inactive (30% as against 17.2% of individuals with low/intermediate social isolation) and had a lower daily consumption of fruit and vegetables (28.5% against 22.1% of individuals with low/intermediate social isolation levels).

**Table 1:** Characteristics of the study population according to social isolation level

	Social Isolation		***t***/χ2	***p***-value	Cohen’s d/ phi
	Low/intermediate (***N*** = 55,559)	High (***N*** = 11,614)			
**Age, mean (SD)**	65.1 (10.3)	70.1 (11.9)	−48.78	< 0.001	0.50 (medium effect size)
**Gender (%)**			1457.93	< 0.001	−0.15 (small effect size)
Female	50.3	67.6			
Male	49.7	32.4			
**Education (%)**			1145.56	< 0.001	0.13 (small effect size)
Primary or less	38.3	54.3			
Secondary	36.5	32.3			
Post-secondary	25.2	13.4			
**Income (%)**			292.93	< 0.001	0.07
Low	31.9	39.4			
Medium	33.1	28.9			
High	35.0	31.8			
**Physical health, mean (SD)**	0.04 (0.7)	−0.22 (0.7)	38.94	< 0.001	0.40 (small effect size)
**Depression (Euro-D) (%)**			1251.69	< 0.001	0.14 (small effect size)*
No	73.3	56.9			
Yes	26.7	43.1			
**Seen/talked to medical doctor in the last 12 months, mean (SD)**	6.7 (9.2)	7.7 (9.8)	−9.90	< 0.001	−0.10
**Excessive alcohol consumption (%)**			29.55	< 0.001	−0.02
No	95.8	96.8			
Yes	4.2	3.2			
**Ever smoked**			107.06	< 0.001	−0.04
No	53.0	56.6			
Yes	47.0	43.4			
**Physical inactivity (%)**			922.52	< 0.001	0.12 (small effect size)
No	82.8	70.0			
Yes	17.2	30.0			
**Not consume fruits or vegetables** **every day (%)**			228.47	< 0.001	0.06
No	77.9	71.5			
Yes	22.1	28.5			

Source: SHARE, wave 6, release 7.0.0., N = 67,173

Notes: SD: Standard Deviation; Tests for two-group comparison (i.e., T test for independent samples (t); chi-square tests (X^2^)); Tests for effect size: Cohen’s d: small effect (≥ 0.20); medium effect (≥ 0.50); large effect (≥ 0.80); Phi: small effect (≥ 0.10); medium effect (≥ 0.30);large effect (≥ 0.50)

Nonetheless, when considering effect size, which measures the magnitude of the differences found, only age, gender, education, physical health, depression and physical inactivity were considered significant.

**Fig. 2:**
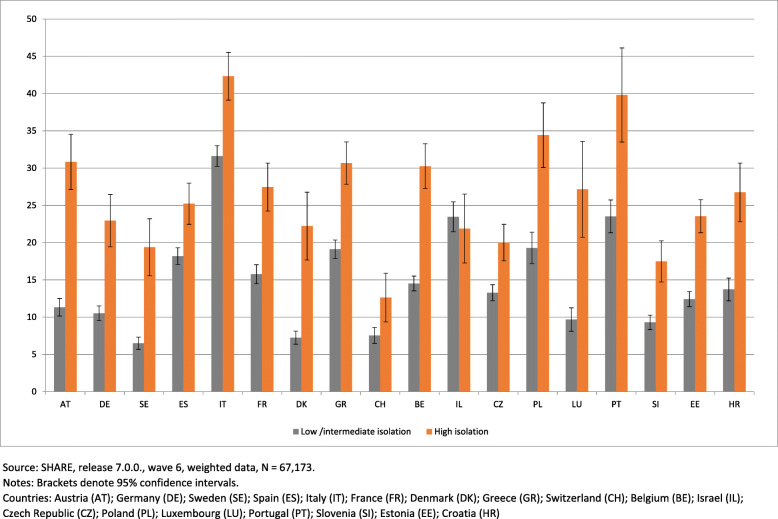
Prevalence of physical inactivity according to social isolation level, by country

Figure 2 shows the prevalence of physical inactivity in the middle-aged and older adults according to social isolation level, by country. Physically inactive individuals have higher levels of social isolation in all countries, except Israel. Italy (42.3%), Portugal (39.8%) and Poland (34.4%) are the countries with higher percentages of physically inactive individuals who experience high social isolation. In several countries, the percentage of individuals who are physically inactive and highly socially isolated is much higher than the percentage of individuals who are physically inactive but not highly socially isolated. This is the case in Austria (30.8% compared to 11.3%), Germany (22.9% compared to 10.5%), Sweden (19.4% compared to 6.5%), France (27.5% compared to 15.8%), Denmark (22.2% compared to 7.2%), Greece (30.7% compared to 19.1%), Belgium (30.3% compared to 14.5%), Luxembourg (27.2% compared to 9.7%), Estonia (23.5% compared to 12.4%) and Croatia (26.7% compared to 13.7%).

**Fig. 3:**
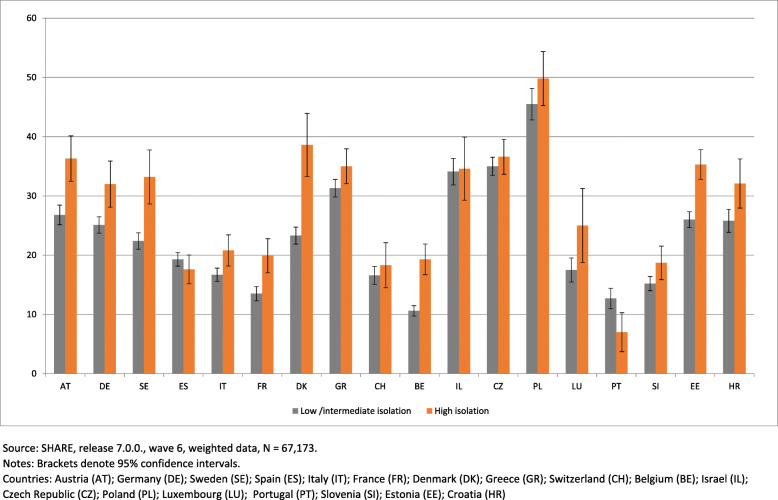
Prevalence of non-consumption of fruit or vegetables on a daily basis, according to social isolation level, by country

Figure 3 shows the prevalence of the non-consumption of fruit or vegetables on a daily basis, according to social isolation level, by country. It highlights that in Austria, Germany, Sweden, Italy, France, Denmark, Belgium, Estonia and Croatia, non-consumption of fruit and vegetables on a daily basis is higher in the highly socially isolated group than in the other group. Nevertheless, in other countries (Spain, Greece, Switzerland, Israel, Czech Republic, Poland, Luxembourg and Slovenia), no differences were found between the two groups.

By contrast, in Portugal, the prevalence of daily non-consumption of fruit or vegetables is higher in the group that is low/intermediate socially isolated.

Table 2 presents the results of adjusted logistic regressions by country, performed in order to test the relationship between social isolation, on the one hand, and physical inactivity (Model 1) and the non-consumption of fruit or vegetables every day (Model 2), on the other hand. The analyses show that high social isolation is significantly associated with physical inactivity in Austria (OR = 2.09, 95% CI 1.63 to 2.69), Germany (OR = 1.40, 95% CI 1.07 to 1.83), Sweden (OR = 1.53, 95% CI 1.12 to 2.10), Denmark (OR = 1.59, 95% CI 1.13 to 2.23), Greece (OR = 1.41, 95% CI 1.18 to 1.70), Belgium (OR = 1.28, 95% CI 1.05 to 1.56), Luxembourg (OR = 1.76, 95% CI 1.14 to 2.71) and Estonia (OR = 1.27, 95% CI 1.06 to 1.51). Thus, highly socially isolated individuals from these countries have an increased likelihood of being physically inactive. In Poland (OR = 1.31, 95% CI 0.99 to 1.75), highly socially isolated individuals have a marginal likelihood of having this health risk behaviour.

**Table 2:** Multivariate logistic regression for high social isolation, by country

Health risk behaviours	Physical inactivity			No fruits or vegetables		
Country	OR	CI 95%	p	OR	CI 95%	p
**Austria**	2.09	(1.63–2.69)	**< 0.001**	1.65	(1.35–2.03)	**< 0.001**
**Germany**	1.40	(1.07–1.83)	**0.013**	1.53	(1.24–1.88)	**< 0.001**
**Sweden**	1.53	(1.12–2.10)	**0.008**	1.69	(1.33–2.15)	**< 0.001**
**Spain**	0.86	(0.71–1.04)	0.123	1.13	(0.95–1.35)	0.170
**Italy**	1.12	(0.95–1.32)	0.178	1.40	(1.15–1.70)	**< 0.001**
**France**	1.19	(0.96–1.48)	0.115	1.82	(1.43–2.32)	**< 0.001**
**Denmark**	1.59	(1.13–2.23)	**0.008**	1.88	(1.44–2.44)	**< 0.001**
**Greece**	1.41	(1.18–1.70)	**< 0.001**	1.17	(1.00–1.37)	**0.051**
**Switzerland**	1.06	(0.73–1.53)	0.758	1.71	(1.26–2.34)	**< 0.001**
**Belgium**	1.28	(1.05–1.56)	**0.013**	2.37	(1.94–2.90)	**< 0.001**
**Israel**	0.97	(0.71–1.32)	0.844	1.19	(0.90–1.58)	0.222
**Czech Republic**	0.99	(0.82–1.21)	0.944	1.31	(1.12–1.53)	**< 0.001**
**Poland**	1.31	(0.99–1.75)	0.063	1.13	(0.90–1.42)	0.303
**Luxembourg**	1.76	(1.14–2.71)	**0.010**	1.77	(1.18–2.65)	**0.006**
**Portugal**	1.09	(0.78–1.54)	0.607	1.25	(0.81–1.94)	0.317
**Slovenia**	0.95	(0.72–1.24)	0.693	1.46	(1.15–1.84)	**0.002**
**Estonia**	1.27	(1.06–1.51)	**0.009**	1.48	(1.29–1.71)	**< 0.001**
**Croatia**	1.26	(0.93–1.71)	0.140	1.56	(1.22–1.99)	**< 0.001**

Source: SHARE, wave 6, release 7.0.0., *N* = 66,963. Model 1: adjusted for age, sex, education, income, physical and mental health, excessive alcohol consumption, ever smoked, number of doctor’s appointments in the last month and non-consumption of fruit or vegetables every day. Model 2: adjusted to variables of Model 1, except non-consumption of fruit or vegetables every day, and plus physical inactivity

Furthermore, the results show that highly socially isolated individuals are more likely to not consume fruit or vegetables every day, than their peers who are low/intermediate socially isolated: Austria (OR = 1.65, 95% CI 1.35 to 2.03), Germany (OR = 1.53, 95% CI 1.24 to 1.88), Sweden (OR = 1.69, 95% CI 1.33 to 2.15), Italy (OR = 1.40, 95% CI 1.15 to 1.70), France (OR = 1.82, 95% CI 1.43 to 2.32), Denmark (OR = 1.88, 95% CI 1.44 to 2.44), Switzerland (OR = 1.71 95% CI 1.26 to 2.34), Belgium (OR = 2.37, 95% CI 1.94 to 2.90), Czech Republic (OR = 1.31, 95% CI 1.12 to 1.53), Luxembourg (OR = 1.77, 95% CI 1.18 to 2.65), Slovenia (OR = 1.46, 95% CI 1.15 to 1.84), Estonia (OR = 1.48, 95% CI 1.29 to 1.71) and Croatia (OR = 1.56, 95% CI 1.22 to 1.99). In Greece (OR = 1.17, 95% CI 1.00 to 1.37), highly socially isolated individuals have a marginal likelihood of not consuming fruit or vegetables every day.

## Discussion

The social isolation of older adults is a growing public health concern, as it has been associated with poor health and premature mortality. This study aims to contribute to filling a gap in the research by focusing on an analysis of the relationship between social isolation and physical inactivity and the non-consumption of fruit or vegetables every day, by comparing 17 European countries plus Israel.

Our results point out that, for the majority of the countries analysed, highly socially isolated individuals are more likely than low/intermediate socially isolated ones to be physically inactive and to consume less fruit or vegetables on a daily basis. In fact, in half of the European countries (Austria, Germany, Sweden, Denmark, Greece, Belgium, Poland, Luxembourg and Estonia) highly socially isolated individuals are more likely to be physically inactive. Previous findings in England, Switzerland and the United States also corroborate this association, since socially isolated older adults were found to engage in less physical activity [4, 14, 52].

Furthermore, our results show that in 14 European countries (Austria, Germany, Sweden, Italy, France, Denmark, Greece, Switzerland, Belgium, Czech Republic, Luxembourg, Slovenia, Estonia, and Croatia), experiencing high levels of social isolation increases the likelihood of having an inadequate diet. In Europe, Kalousova investigated whether socially isolated older adults (65+) living in Eastern Europe (Czech Republic, Poland, and Hungary) experienced an increased risk of dietary inadequacy. The author [32] found that social isolation was only associated with a lower likelihood of having a daily serving of fruits or vegetables among Czech and Polish older adults. The same result was found in the Swiss population, with socially isolated people, independent of their age, reporting a higher risk of poor diet [14].

Even though no European region patterns were found in our analysis, this study emphasised the association between social isolation, and physical inactivity and inadequate diet in the majority of European countries.

Despite previous studies on specific countries that stressed the association between social isolation, and physical inactivity and inadequate diet, the mechanisms behind these associations are still weakly understood. Rook [53] suggests that, in order to understand what socially isolated individuals lack that makes them vulnerable in terms of health problems, one must take into account the content and functions of social exchanges. According to this author, social relationships have three main functions: social integration, social support and companionship. Regarding the first function, socially isolated individuals are less subjected to the normative functions of social relationships [53]. Along the same lines, Umberson [54] highlights that relationships act directly and indirectly on health risk behaviours. In our study, this direct path would operate through social control of physical activity and diet behaviour. The indirect path occurs under a sense of obligation, meaning and life purpose of the relationships [54]. People would avoid physical inactivity and poor diet, because of feeling obliged towards others or to preserve their social roles, for example.

Social support, the second function of social relationships, can ensure a better diet and physical activity, particularly for those who have trouble with instrumental activities of daily life. In fact, social support can ensure the purchase of food and preparation of meals, and physical exercise, when these activities cannot be carried out independently.

Finally, the third function of social relationships (companionship) implies pleasant interactions and joint activities with others that create well-being. Undertaking physical activities with others might encourage physical exercise and sharing meals may provide opportunities to have a better diet. The above-mentioned explanatory mechanisms need to be further explored.

As in previous studies, we also found that socially isolated individuals are older [9, 14, 55], less educated [14, 55, 56] and have a lower income [55–57]. Furthermore, in our research, socially isolated individuals are predominantly women but, in the scientific literature, the findings regarding gender differences are mixed. While Steptoe et al. [55] found no gender differences in social isolation, Menec et al. [9] and Cudjoe et al. [56] concluded that being male is associated with higher odds of being socially isolated and Szaflarski [58] states that women are more socially isolated than men, due to their greater involvement in housework and care responsibilities. Further studies should be conducted to clarify these differences.

Concerning health, our findings are in line with earlier research, indicating that socially isolated individuals have worse physical and mental health, a higher number of visits to the doctor [6, 9, 14, 55] and lower percentages of excessive alcohol consumption [59]. Lastly, in our study, the highly socially isolated group has a lower percentage of individuals who smoked. This last finding contradicts previous results by Shankar et al. [60], which concluded that individuals who are socially isolated have a greater risk of smoking. This outcome might be explained by the characteristics of the highly socially isolated group of our sample, since this descriptive result is not adjusted for any confounders.

These findings indicate that attention should be paid to highly socially isolated middle-aged and older adults, as they are more prone to physical inactivity and a lower intake of fruit or vegetables, which can be harmful for one’s health. According to the WHO (World Health Organization) [61], physical inactivity and an unhealthy diet are two of the four main behavioural risk factors for non-communicable diseases. As stated by Schrempft et al. [4], the persistent lack of physical activity in socially isolated individuals will contribute to an increased likelihood of chronic diseases and disability in older people. Besides being a risk factor for non-communicable diseases, such as several forms of cancer, diabetes, hypertension, coronary and cerebrovascular diseases, overweight/obesity and all-cause mortality [62], insufficient physical activity also has a negative effect on mental health and quality of life [63].

Additionally, the insufficient consumption of fruit and vegetables causes gastrointestinal cancer deaths, ischaemic heart disease deaths and stroke deaths globally [64]. By contrast, the intake of fruit and vegetables was found to improve physical, mental and cognitive health [24].

Therefore, older people, their families, social and medical institutions, healthcare professionals and country policy makers should be aware of physical inactivity and inadequate diet associated with the absence of social interaction, contacts and relationships and implement actions in order to mitigate/overcome this situation. For that reason, in an ageing society, identifying this risk group and its awareness of healthy behaviours are of the utmost importance.

### Limitations

The results of this study may have been affected by the fact that definitions of “fruit” and “vegetables” vary from country to country [65]. Therefore, to obtain more accurate results, the design of a common European classification of food is recommended. Furthermore, regarding the physical inactivity measure, the SHARE data only enables us to find out how often the respondents engaged in moderate or vigorous activities, in general. SHARE does not provide information about the type of activities in which individuals were involved and the time spent on them, not allowing the adoption of a measure that follows the WHO recommendation [66]. Access to this kind of information would enable us to obtain more precise and comparative analyses with non-SHARE countries. Finally, as it is a cross-sectional study, we cannot assume causality.

## Conclusions

This is the first study to analyse, on a European level, the relationship between social isolation and physical inactivity and inadequate diet. The main results show that, in the majority of the countries analysed, socially isolated individuals are more prone to be physically inactive and to consume less fruit or vegetables on a daily basis compared with non-isolated ones. Overall, our results reinforce the need for public social and health policies targeted towards European socially isolated middle-aged and older adults. Policies should counter social isolation by creating opportunities for social interaction or, at least, should reduce the effect of social isolation through social support capable of promoting opportunities for engaging in physical activity and having a balanced diet. More age-friendly environments are required to tackle social isolation among older individuals.

## Data Availability

This paper uses data from SHARE Waves 4 and 6 release 6.1.0 of 28 March 2018 (DOIs: 10.6103/SHARE.w4.610 and 10.6103/SHARE.w6.610). The SHARE data are available and can be downloaded from the SHARE Research Data Center under the following conditions: applicants must have a scientific affiliation and sign a statement confirming that under no circumstances will the data be used for other than purely scientific purposes. Data will only be made available after these documents have been received by mail or fax (care of Josette Janssen; address: CentERdata, Tilburg University, P.O. Box 90153, 5000 LE Tilburg, The Netherlands; e-mail: jjanssen@uvt.nl. Methodological details of the SHARE study design and data collection are presented elsewhere (Börsch-Supan et al., 2013).

## Abbreviations

CFA: Confirmatory Factor Analysis
CFI: Comparative Fit Index
CI: Confidence Intervals
ISCED: International Standard Classification of Education
OECD: Organisation for Economic Co-operation and Development
OR: Odds Ratio
RMSEA: Root Mean Square Error of Approximation
SHARE: Survey of Health, Ageing and Retirement in Europe
TLI: Tucker-Lewis Index
2SRI: Two Stage Residual Inclusion
WHO: World Health Organization
